# Development and validation of a ^18^F-FDG PET/CT radiomics nomogram for predicting progression free survival in locally advanced cervical cancer: a retrospective multicenter study

**DOI:** 10.1186/s12885-024-11917-3

**Published:** 2024-01-30

**Authors:** Huiling Liu, Yongbin Cui, Cheng Chang, Zichun Zhou, Yalin Zhang, Changsheng Ma, Yong Yin, Ruozheng Wang

**Affiliations:** 1 Department of Radiation Oncology, The Third Affillated Teaching Hospital of Xinjiang Medical University, Affilated Cancer Hospital, Urumuqi, China; 2 Department of Radiation Oncology, Shandong Cancer Hospital and Institute, Shandong First Medical University, Shandong Academy of Medical Sciences, Jinan, China; 3 Department of Nuclear Medicine, Third Affiliated Hospital of Xinjiang Medical University, State Key Laboratory of Pathogenesis, Prevention and Treatment of High Incidence Diseases in Central Asia, Urumqi, China; 4 School of Mechanical, Electrical and Information Engineering, Shandong University, Weihai, China; 5 Xinjiang Key Laboratory of Oncology, Urumqi, China; 6 Key Laboratory of Cancer Immunotherapy and Radiotherapy, Chinese Academy of Medical Sciences, Urumqi, China

**Keywords:** Locally advanced cervical cancer, ^18^F-FDG PET/CT, Radiomics, Prediction model, Machine learning, Progression free survival

## Abstract

**Background:**

The existing staging system cannot meet the needs of accurate survival prediction. Accurate survival prediction for locally advanced cervical cancer (LACC) patients who have undergone concurrent radiochemotherapy (CCRT) can improve their treatment management. Thus, this present study aimed to develop and validate radiomics models based on pretreatment ^18^Fluorine-fluorodeoxyglucose (^18^F-FDG) positron emission tomography (PET)-computed tomography (CT) images to accurately predict the prognosis in patients.

**Methods:**

The data from 190 consecutive patients with LACC who underwent pretreatment ^18^F-FDG PET-CT and CCRT at two cancer hospitals were retrospectively analyzed; 176 patients from the same hospital were randomly divided into training (*n* = 117) and internal validation (*n* = 50) cohorts. Clinical features were selected from the training cohort using univariate and multivariate Cox proportional hazards models; radiomic features were extracted from PET and CT images and filtered using least absolute shrinkage and selection operator and Cox proportional hazard regression. Three prediction models and a nomogram were then constructed using the previously selected clinical, CT and PET radiomics features. The external validation cohort that was used to validate the models included 23 patients with LACC from another cancer hospital. The predictive performance of the constructed models was evaluated using receiver operator characteristic curves, Kaplan Meier curves, and a nomogram.

**Results:**

In total, one clinical, one PET radiomics, and three CT radiomics features were significantly associated with progression-free survival in the training cohort. Across all three cohorts, the combined model displayed better efficacy and clinical utility than any of these parameters alone in predicting 3-year progression-free survival (area under curve: 0.661, 0.718, and 0.775; C-index: 0.698, 0.724, and 0.705, respectively) and 5-year progression-free survival (area under curve: 0.661, 0.711, and 0.767; C-index, 0.698, 0.722, and 0.676, respectively). On subsequent construction of a nomogram, the calibration curve demonstrated good agreement between actually observed and nomogram-predicted values.

**Conclusions:**

In this study, a clinico-radiomics prediction model was developed and successfully validated using an independent external validation cohort. The nomogram incorporating radiomics and clinical features could be a useful clinical tool for the early and accurate assessment of long-term prognosis in patients with LACC patients who undergo concurrent chemoradiotherapy.

**Supplementary Information:**

The online version contains supplementary material available at 10.1186/s12885-024-11917-3.

## Introduction

Cervical cancer is the fourth most frequent cause of cancer-related morbidity and mortality in women worldwide, especially in developing countries and low/middle-income areas [1]. In contrast to the global trends of decrease in cervical cancer incidence, young women in China are showing a substantial increase [2]. Approximately 40 to 50% of patients are initially diagnosed with locally advanced cervical cancer (LACC) and approximately 6% are found to have primary metastatic disease, which is the principal cause of death [3]. In this context, the International Federation of Obstetrics and Gynecology (FIGO) system has included lymph node status in the staging classification in 2018; LACC includes stages IB to IVA tumors [4]. International treatment guidelines recommend platinum-based concurrent chemoradiotherapy (CCRT) as the standard treatment for LACC [5, 6]. Notably, the 5-year overall survival (OS) for locally advanced and metastatic cervical cancer is estimated to be approximately 65% and 17%, respectively [7]. In addition, approximately 35% of patients with LACC experience relapse and the prognosis is poor, as median survival after recurrence extends to approximately 10–12 months [8]. It is therefore essential to accurately identify patients who are at high risk of progression and develop better treatment regimens for LACC.

Current clinically relevant and evidence-based guidelines mainly suggest use of the FIGO staging system for selection of the treatment regimen and prediction of prognosis [9]. This system has been revised periodically based on clinical risk factors to improve staging and differentiation of prognostic outcomes [10]. In this context, conventional medical images only provide information related to the tumor structure and facilitate diagnosis; however, they cannot help predict therapeutic responses and future prognosis [11]. As patients with cervical cancer having the same FIGO stage have different clinical outcomes, this staging system does not fully meet the needs for prediction of clinical prognosis [12]. There is therefore an urgent need to find novel non-invasive biomarkers that can provide better pre-treatment information regarding tumor heterogeneity; this may in turn help clinicians personalize treatment schedules.

Radiomics extracts quantitative data from medical images to generate imaging biomarkers, which demonstrate tumor spatial and temporal heterogeneity; this provides a support tool for decision-making in clinical practice [13]. Magnetic resonance imaging-based radiomics has been reported to be useful in distinguishing clinico-pathological characteristics and predicting prognosis in cervical cancer [14, 15]. In addition, ^18^Fluorine-fluorodeoxyglucose positron emission tomography (^18^F-FDG PET)-based radiomics has been used to predict the tumor stage and treatment outcomes in these patients [16, 17]. However, few studies using PET/computed tomography (CT)-based radiomics have aimed to predict prognosis in cervical cancer [18, 19]; none have employed independent validation in the clinical setting.

Therefore, we aimed to develop prediction models and a visually quantitative nomogram (which incorporated clinical features, PET metabolic parameters, and PET and CT radiomics features) in order to predict 3-year and 5-year progression-free survival (PFS) in patients with LACC who received CCRT. The potential benefits of individualized prediction performance were further validated in an independent dataset.

## Materials and methods

### Study design and workflow

**Fig. 1:**
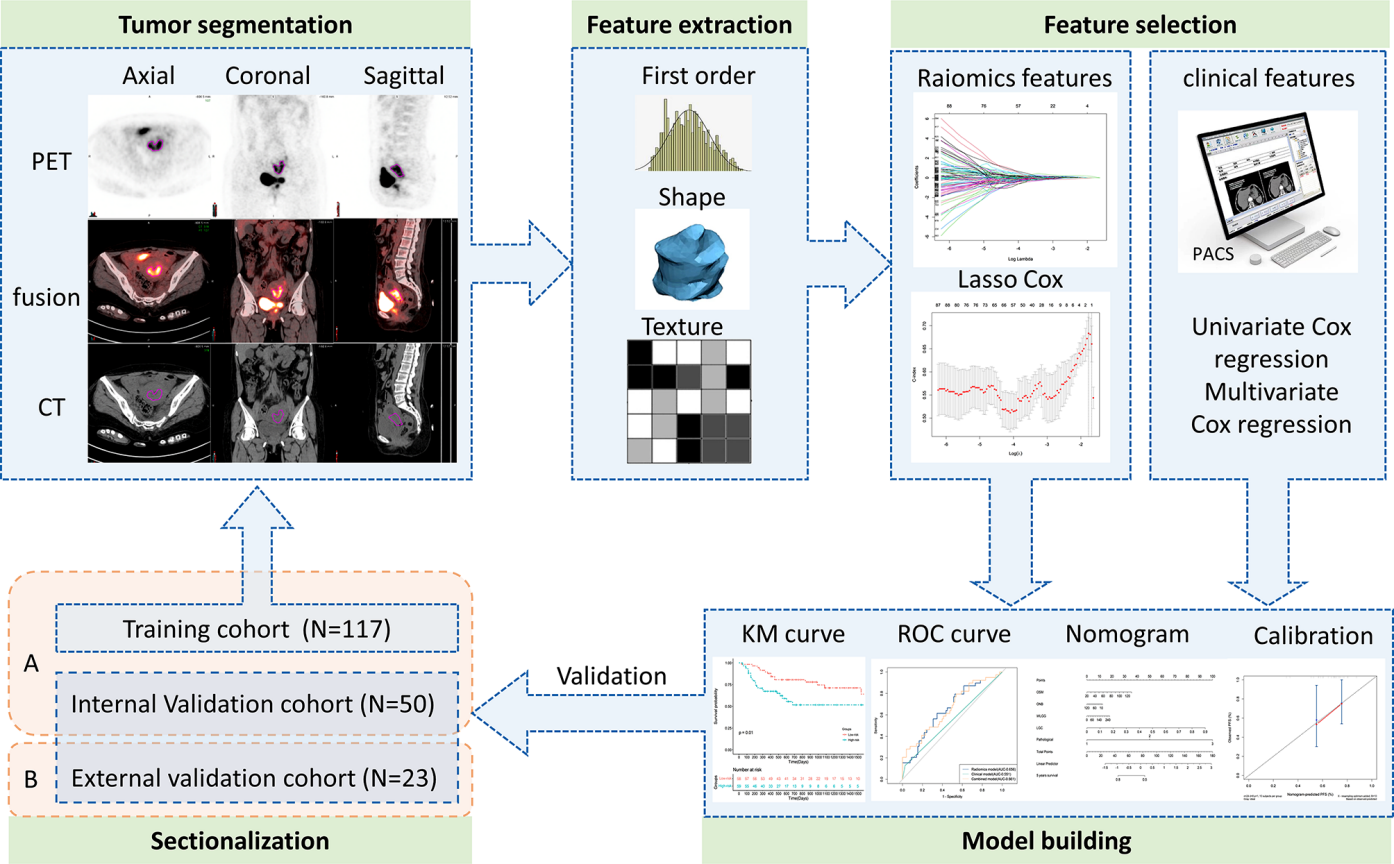
The study design and workflow

The study design and workflow have been illustrated in Fig. 1. Patients who had been pathologically diagnosed with LACC and received CCRT were recruited in the present study and their PET/CT images were obtained for radiomics analysis. The radiomic features were extracted and selected based on their clinical effectiveness in predicting 3- and 5-year PFS.

### Patients

This multicenter retrospective study received ethical approval from the Xinjiang Medical University Affiliated Cancer Hospital and Shandong First Medical University Affiliated Cancer Hospital. The study was performed in accordance with the principles of the Declaration of Helsinki (as revised in 2013); the need for informed consent was waived.

**Fig. 2:**
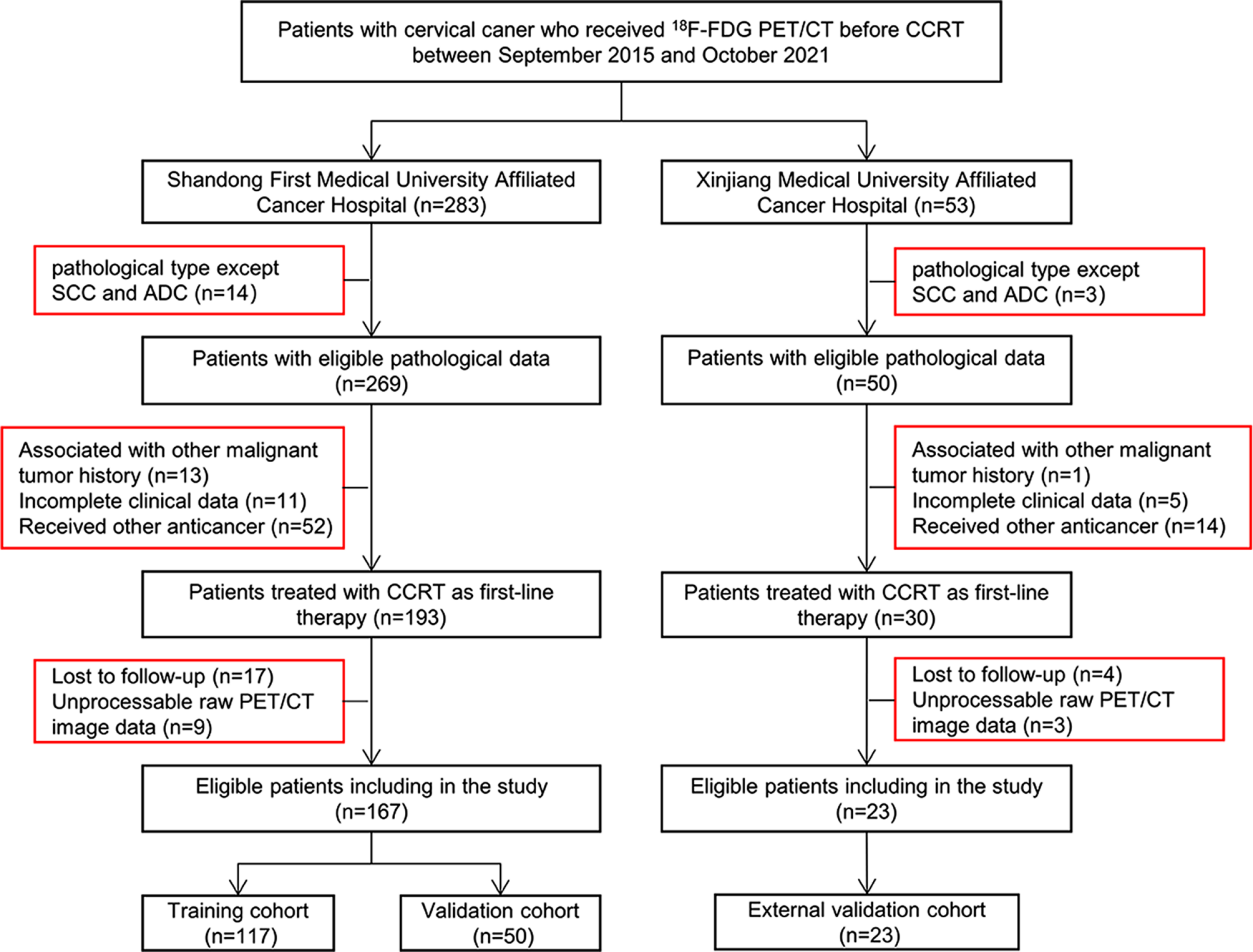
Flowchart showing the patient selection and exclusion in this study

This study included 190 consecutive patients who were pathologically diagnosed with LACC and had received CCRT at two tumor hospitals (Shandong First Medical University Affiliated Cancer Hospital and Xinjiang Medical University Affiliated Cancer Hospital) between September 2015 and October 2021. Patients fulfilling the following criteria were included: (1) having pathologically diagnosed primary cervical cancer, (2) having a tumor of stage IB3-IVA (after restaging by a gynecological oncologist with 10 years of experience according to the 2018 FIGO staging criteria), (3) having complete clinical data that could be retrieved from the electronic medical records, and (4) having pretreatment standard routine whole-body ^18^F-FDG PET/CT scan. The following patients were excluded: (1) those having pathological types other than squamous cell carcinoma and adenocarcinoma, (2) those having a previous history of another malignant tumor or anticancer treatment prior to the PET/CT scan, (3) those having incomplete clinical data or non-adherence to follow-up, and (4) those in whom the raw data from the ^18^F-FDG PET/CT images could not be processed. The patient recruitment process has been shown in Fig. 2.

Finally, a total of 167 eligible patients from the Shandong First Medical University Affiliated Cancer Hospital were enrolled and randomly divided into training (*n* = 117) and internal validation (*n* = 50) cohorts in a 7:3 ratio. A total of 23 patients treated at the Xinjiang Medical University Affiliated Cancer Hospital were considered eligible and were included in the external validation cohort. The baseline clinical data including age; FIGO stage; pathological type; history of abortion; menopausal status; the maximum tumor diameter (MTD); presence of lymph node metastasis (LNM); treatment regimens; total external beam radiotherapy dose; chemotherapy regimen; and lymphocyte, monocyte, neutrophil, and platelet counts were obtained from the electronic medical records. The distribution of clinical characteristics and PET metabolic parameters between the training and internal validation cohorts was found to be balanced (Table 1).

**Table 1:** Patient characteristics and PET metabolic parameters in the training, internal validation and external validation cohort

	Training cohort (*n* = 117)	Internal Validation cohort (*n* = 50)	P values	External Validation cohort (*n* = 23)
Age (years)	53.93 ± 10.11	53.40 ± 9.90	0.753	50.65 ± 9.34
Age			0.672	
<55 years	59 (50.43%)	27 (54.00%)		16 (69.56%)
≥55 years	58 (49.57%)	23 (46.00%)		7 (30.43%)
Abortion			0.426	
No	65 (55.56%)	25 (50.00%)		10 (43.48%)
Yes	52 (44.44%)	25 (50.00%)		13 (56.52%)
Menstruation status			0.746	
Premenopause	46 (39.32%)	21 (42.00%)		14 (60.87%)
Menopause	71 (60.68%)	29 (58.00%)		9 (39.13%)
Pathology			0.997	
SCC	110 (94.02%)	47 (94.00%)		19 (82.61%)
ADC	7 (5.98%)	3 (6.00%)		4 (17.39%)
FIGO stage			0.169	
IB3	5 (4.27%)	1 (2.00%)		0 (0%)
IIA - IIB	18 (15.39%)	8 (16.00%)		8 (34.78%)
IIIA - IIIC	90 (76.92%)	35 (70.00%)		11 (47.83%)
IVA	4 (3.42%)	6 (12.00%)		4 (17.39%)
MTD (cm)			0.208	
<4.25	37 (31.62%)	11 (22.00%)		9 (39.13%)
≥4.25	80 (68.38%)	39 (78.00%)		14 (60.87%)
LNM			0.527	
N 0	41 (35.04%)	15 (30.00%)		9 (39.13%)
N +	76 (64.96%)	35 (70.00%)		14 (60.87%)
EBRT total dose (Gy)			0.890	
<50.4	34 (29.06%)	14 (28.00%)		4 (17.39%)
≥50.4	83 (70.94%)	36 (72.00%)		19 (82.61%)
Chemotherapy regimen			0.709	
Cisplatin	5 (4.27%)	3 (6.00%)		16 (69.56%)
Carboplatin	65 (55.56%)	30 (60.00%)		1 (4.35%)
Nedaplatin	47 (40.17%)	17 (34.00%)		6 (26.09%)
Chemotherapy cycle			0.673	
3	39 (33.33%)	15 (30.00%)		1 (4.35%)
>3	78 (66.67%)	35 (70.00%)		22 (95.65%)
LMR			0.734	
<3.25	43 (36.75%)	17 (34.00%)		5 (21.74%)
≥3.25	74 (63.25%)	33 (66.00%)		18 (78.26%)
NLR			0.645	
<3	70 (59.83%)	28 (56.00%)		22 (95.65%)
≥3	47 (40.17%)	22 (44.00%)		1 (4.35%)
PLR			0.491	
<150	38 (32.48%)	19 (38.00%)		16 (69.57%)
≥150	79 (67.52%)	31 (62.00%)		7 (30.43%)
MTV	31.68 (15.10,52.36)	35.65 (18.92,70.44)	0.270	11.01 (6.56,26.34)
TLG	286.18 (130.08,576.00)	330.40 (150.08,706.63)	0.392	101.02 (57.04,180.98)
SUVmax	15.00 (11.69,21.36)	14.37 (11.87,18.35)	0.316	14.11 (9.75,17.54)
SUVmean	8.91 (6.91,12.08)	8.39 (7.13,10.92)	0.475	8.55 (5.54,11.00)
SUVmin	2.75 (2.32,3.44)	2.59 (1.71,3.04)	0.065	3.54 (2.60,4.53)

SCC: squamous cell carcinoma; ADC: adenocarcinoma; FIGO: International Federation of Gynecology and Obstetrics; EBRT: external beam radiotherapy; MTD: the maximum tumor diameter; LNM: lymph nodes metastasis; LMR: lymphocyte-to-monocyte ratio; NLR: neutrophil-to-lymphocyte ratio; PLR: platelet-to-lymphocyte ratio; MTV: metabolic tumor volume; TLG: total lesion glycolysis; SUVmax: maximum standardized uptake value; SUVmean: mean standard uptake value; SUVmin: minimum standardized uptake value

### Treatment and follow-up

All patients received platinum-based chemotherapy in combination with image-guided external beam radiotherapy and brachytherapy up to a total dose of 85–90 Gy. External beam radiotherapy was delivered at a dose of 1.8-2.0 Gy/fraction, up to a dose of 45–50 Gy. Positive pelvic lymph nodes were simultaneously boosted with an additional dose of 10–20 Gy. The platinum-based chemotherapy regimen included cisplatin, carboplatin, and nedaplatin in 5, 65, and 47 patients, respectively. Patients were followed-up regularly every 3–6 months during the first two years after CCRT, twice a year during years 3–5, and once a year thereafter. The clinical outcome events included first local recurrence, lymph node metastasis, distant metastasis, and death. In the present study, PFS was defined by the interval between the end of CCRT and first occurrence of the endpoint event or October 30, 2022. Disease progression was confirmed by gynecological examination, imaging, or biopsy.

### PET-CT image acquisition

Baseline PET/CT examination was performed within 2 weeks before biopsy and CCRT. During the study, PET/CT images was acquired using two whole-body PET/CT scanners; the Philips Gemini TF (Phillips Medical Systems, Holland) was used at the Shandong First Medical University Affiliated Cancer Hospital and Philips ingenuity TF (Phillips Medical Systems, Holland) was used at the Xinjiang Medical University Affiliated Cancer Hospital. The patients fasted for more than 6 h, and their blood glucose levels were measured to ensure a level of < 140 mg/dL. The patients received ^18^F-FDG intravenously at a dose of 4.4 MBq/kg; whole-body PET and CT scans were performed one hour later. Spiral CT scans (dose modulation with a quality reference of 150 mAs, 130 kV, a 512 × 512 matrix, and 3-mm slice thickness) were performed immediately prior to the PET scans (1 min in each bed; 144 × 144 matrix); images were acquired from the distal femur to the top of the skull. The PET images were attenuated, corrected, and reconstructed using an iterative ordered subset expectation maximization method. The PET images were then fused with CT images to obtain whole-body transverse, coronal, and sagittal images. All images were acquired using the respiratory gating technique.

### Tumor segmentation

**Fig. 3:**
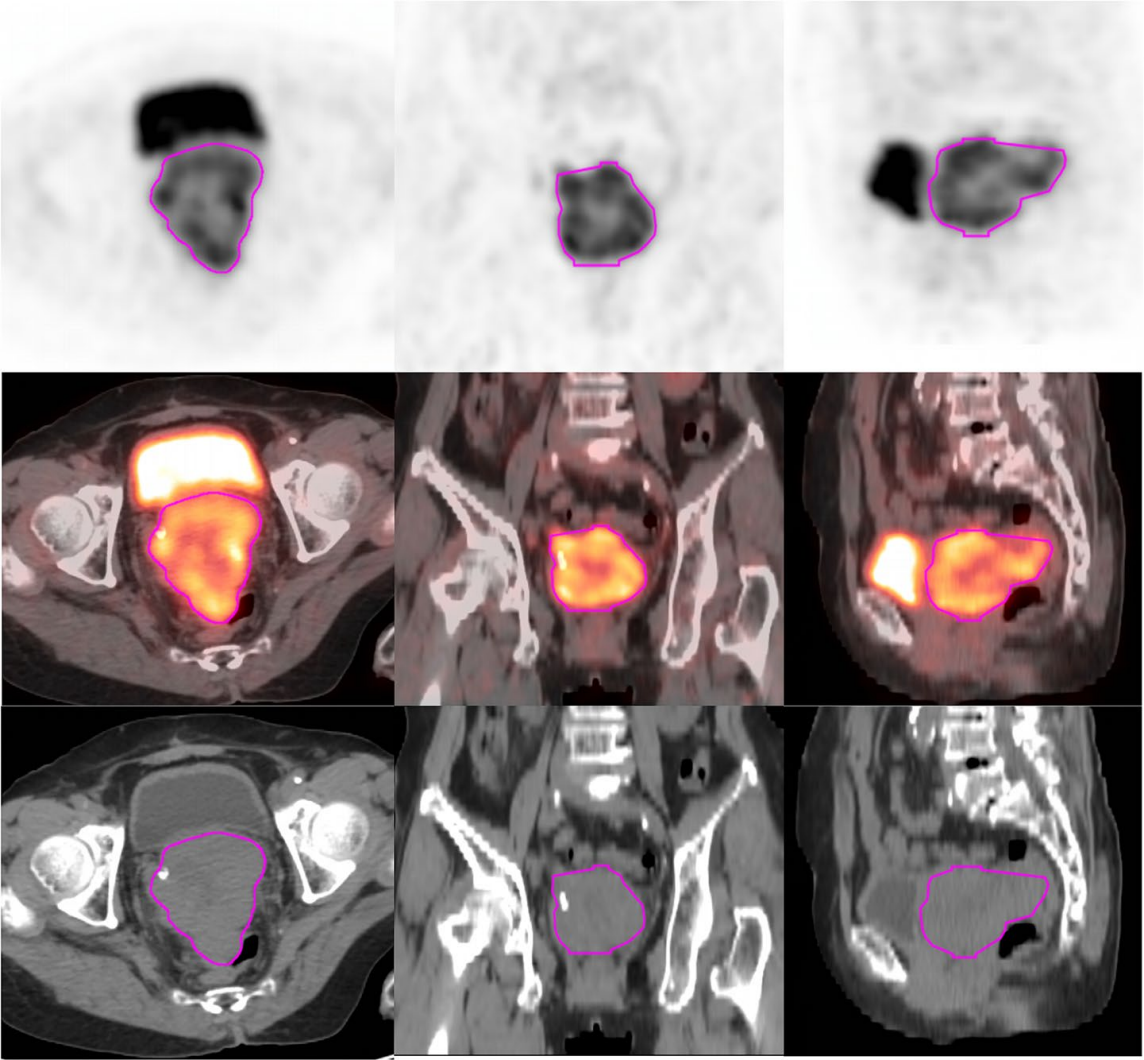
Representative example of the regions of interest (ROI) segmentation on axial, coronal, and sagittal PET/CT

The regions of interest (ROIs) were delineated using the MIM Maestro version 7.1.7 (MIM Software Inc., Cleveland, OH, USA) package. An experienced nuclear medicine physician delineated the margins in axial, coronal, and sagittal PET scans to adequately include the primary tumor. An experienced oncologist then used a fixed threshold value of 42% of the maximum standardized uptake value (SUVmax) to automatically segment the ROIs [20]; regions within the bladder were manually excluded from the segmentation results. The metabolic active tumor volume (MTV), mean standardized uptake value (SUVmean), total lesion glycolysis (TLG), and SUVmax for the obtained ROIs were automatically calculated and derived by the MIM Software package. Another experienced oncologist checked and modified the contoured ROIs, slice-by-slice, and separately transferred them onto PET and CT images using rigid registration. Figure 3 illustrates tumor segmentation in a patient using a fixed percentage threshold-based algorithm.

### Feature extraction

Pre-treatment PET and CT images and the corresponding ROIs were loaded onto AccuContour software version 3.2 (Manteia Medical Technologies Co. Ltd., Xiamen, China), which allows for standardized pre-processing of medical imaging data [20]. This software package was developed using the open-source Python package, Pyradiomics version 3.0.1, which codes with a graphical user interface that allows extraction of most features defined by the Image Biomarker Standardization Initiative [21]. The original PET and CT images were filtered using wavelet, Laplacian-of-Gaussian (sigma = 1.0), square, square root, logarithm, exponential, and gradient filters to generate processed images. The original and processed images were then uploaded onto AccuContour software to extract the radiomics features. The absolute intensity quantization was used and the high bounds was 25. The number of discretization levels were set as 64. The PET radiomics features and CT radiomics features were fused into classification model by late fusion. The extracted features have been listed in Table S2.

### Feature selection and development of prediction models

Effective prognostic radiomics features were selected using a least absolute shrinkage and selection operator Cox model with 10-fold cross-validation; clinical features were selected using univariate and multivariate Cox proportional hazards models (*P* < 0.05) in the training cohort. Radiomics, clinical, and combined models with good prediction performance were then developed separately to predict 3- and 5-year PFS in the training, internal validation, and external validation cohorts.

### Prediction performance and clinical utility of prediction models

Patients in the training cohort were divided into high- and low-risk subgroups using the Kaplan Meier method, and the log-rank test was used to test differences in survival between these subgroups (*P* < 0.05). Prediction performance for the survival rate was evaluated based on the receiver operating characteristics (ROC) curve and C-index. Decision curve analysis (DCA) was used to evaluate the clinical applicability of the prediction models.

### Establishment and validation of the nomogram

An individualized visual nomogram was finally constructed for predicting 3- and 5-year PFS in LACC using the previously selected clinical and PET/CT radiomics features from the training cohort [22]. The concordance between nomogram-predicted and actual PFS was evaluated in all three cohorts using calibration curves.

### Statistical analysis

Radiomic feature extraction was performed using AccuContour software, version 3.2 (Manteia Medical Technologies Co. Ltd., Xiamen, China). All statistical analyses were performed using R software, version 3.4.0 (R Foundation for Statistical Computing, Vienna, Austria) and SPSS, version 25.0 (IBM, Armonk, NY). The optimum cut-off value for the clinical features was defined based on the Youden index obtained from the ROC curve of the training cohort. Comparisons between groups were performed using the *t* or Mann-Whitney U tests and differences between rates were evaluated using χ^2^ or Fisher’s exact tests, as appropriate. All statistical tests were two-sided and *P* values less than 0.05 were considered statistically significant.

## Results

### Patient characteristics ant PET metabolic parameters

A total of 190 patients with LACC were enrolled in this retrospective study. Among them, 167 consecutive patients (157 with squamous cell carcinoma and 10 with adenocarcinoma) were treated at the Shandong First Medical University Affiliated Cancer Hospital. They were divided into training and internal validation cohorts, which included 117 and 50 patients, respectively. The 23 patients from the Xinjiang Medical University Affiliated Cancer Hospital (comprising 19 and 4 patients with squamous cell carcinoma and adenocarcinoma, respectively) served as the external validation cohort. The patient characteristics and PET metabolic parameters from the training, internal validation, and external validation cohorts are summarized and compared in Table 1. There were no significant differences between the training and internal validation cohorts in terms of the variables assessed (*P* > 0.05).

### Feature selection and development of prediction models

A total of 1409 radiomic features were automatically calculated and extracted from each ROI in the PET and CT images; among them, 107, 744, and 93 features were computed from the original, wavelet, and each of the other processed images, respectively. Three CT (Figure S1A, C) and one PET (Figure S1B, D) radiomics features were filtered using the least absolute shrinkage and selection operator (LASSO) Cox model. Table S2 shows the selected PET and CT radiomics features. Radiomics models were then constructed using the selected radiomics features to predict 3- and 5-year PFS. Table 2 shows the results from univariate and multivariate Cox proportional hazards analysis for the clinical factors associated with 3-year and 5-year PFS in the training cohort. The results demonstrated pathological type to be the only independent prognostic predictor for PFS (*P* < 0.05). The combined models for predicting 3- and 5-year PFS were finally developed using the selected radiomics features and pathological type.

**Table 2:** The clinical factors and PET metabolic parameters analysis in the training cohort

	Univariate Cox P-values	Multivariate Cox P-values	HR
Age	0.439		
Abortion	0.956		
Menstruation status	0.449		
Pathology	0.002	0.006	2.862 (0.911–8.987)
FIGO stage	0.014	0.405	
MTD	0.015	0.429	
LNM	0.010	0.452	
EBRT total dose	0.710		
Chemotherapy model	0.089		
Chemotherapy cycle	0.006	0.091	
LMR	0.785		
NLR	0.343		
PLR	0.993		
MTV	0.004	0.072	
TLG	0.043	0.321	
SUVmax	0.906		
SUVmean	0.846		
SUVmin	0.327		

PFS: progress free survival; FIGO: International Federation of Gynecology and Obstetrics; MTD: the maximum tumor diameter; LNM: lymph nodes metastasis; EBRT: external beam radiotherapy; LMR: lymphocyte to monocyte ratio; NLR: neutrophil to lymphocyte ratio; PLR: platelet to lymphocyte ratio; MTV: metabolic tumor volume; TLG: total lesion glycolysis; SUVmax: maximum standardized uptake value; SUVmean: mean standard uptake value; SUVmin: minimum standardized uptake value

### Prediction performance and clinical utility of prediction models

**Fig. 4:**
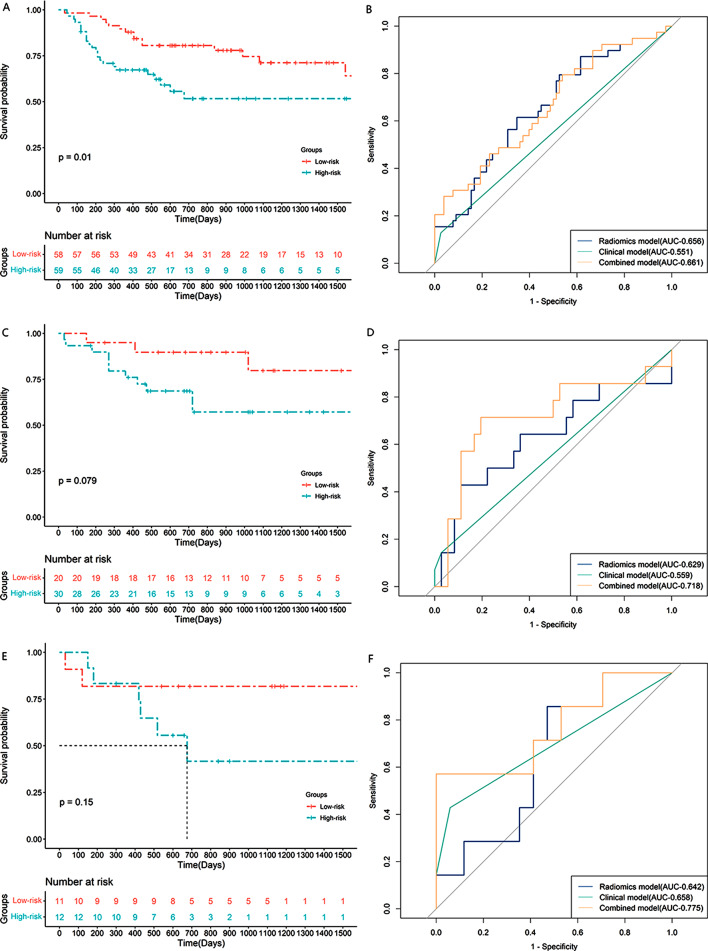
The KM curve in training cohort (**A**), internal validation cohort (**C**) and external validation cohort (**E**). The ROC curve of 3-year PFS prediction model in training cohort (**B**), internal validation cohort (**D**) and external validation cohort (**F**). PFS, progression free survival; KM, Kaplan-Meier; ROC, receiver operator characteristic

As shown in the Kaplan-Meier curves for 3-year PFS (Fig. 4A, C, E), the selected radiomics and clinical features effectively distinguished between the high- and low-risk groups. The ROC curves demonstrated the performance of the 3 prediction models in predicting 3-year PFS (Fig. 4B, D, F). In terms of 3-year PFS prediction, the combined model demonstrated optimal discrimination in the training (area under the curve [AUC] = 0.661, C-index = 0.698), internal validation (AUC = 0.718, C-index = 0.724), and external validation (AUC = 0.775, C-index = 0.705) cohorts (Table S5).

The Kaplan-Meier curves for 5-year PFS (Figure S2A, C, E) also demonstrated similar results. The ROC curves demonstrated the performance of the 3 prediction models in predicting 5-year PFS (Figure S2B, D, F). Among the three models, the combined model demonstrated optimal discrimination and the best values for sensitivity, specificity, and accuracy of prediction in the training (AUC = 0.661, C-index = 0.698), internal validation (AUC = 0.711, C-index = 0.722), and external validation (AUC = 0.767, C-index = 0.676) cohorts; the results are summarized in Table 3.

**Table 3:** Performance of prediction models for predicting 3-year and 5-year PFS in LACC

	3-year PFS			5-year PFS	
	AUC	C-index		AUC	C-index
Training Cohort					
Radiomics model	0.656	0.689		0.656	0.689
Clinical model	0.551	0.556		0.551	0.556
Combined model	0.661	0.698		0.661	0.698
Internal Validation Cohort					
Radiomics model	0.629	0.631		0.632	0.630
Clinical model	0.559	0.557		0.553	0.556
Combined model	0.718	0.724		0.711	0.722
External Validation Cohort					
Radiomics model	0.642	0.619		0.642	0.618
Clinical model	0.658	0.619		0.654	0.608
Combined model	0.775	0.705		0.767	0.676

### Establishment and validation of the nomogram

**Fig. 5:**
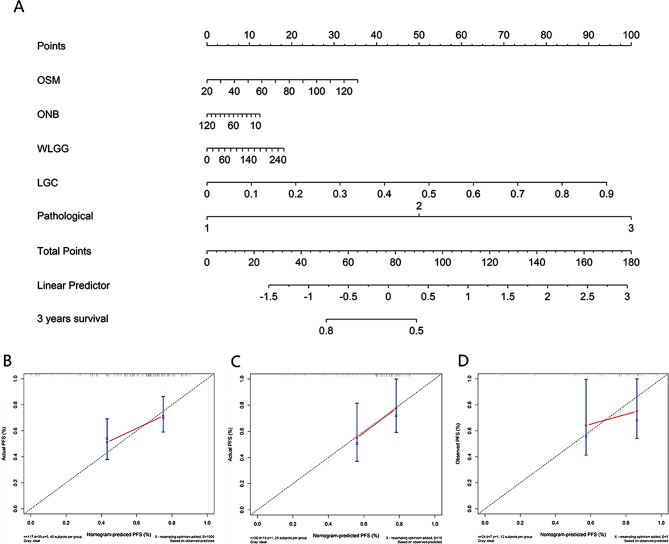
Developed the prediction nomogram based on selected radiomics and clinical features predicting 3-year PFS in training cohort (**A**). The probability of each predictor could be converted into scores according to the first scale “Points” at the top of the nomogram. After adding up the corresponding prediction probability at the bottom of the nomogram was the 3-year PFS. Calibration curves of nomogram in training (**B**), internal validation cohort (**C**) and external validation cohort (**D**), respectively. The X-axis represented the predicted probability estimated by nomogram, whereas the Y-axis represented the actual observed rates. The gray dashed line represented a perfect prediction by an ideal model, and the pink solid line represented the apparent prediction of nomogram. Calibration curves showed the actual probability corresponded closely to the prediction of nomogram. OSM: original, shape, Maximum2DDiameterRow; ONB: original, ngtdm, Busyness; WLGG: wavelet-LLL, glszm, GrayLevelNonUniformity; LGC: logarithm, glcm, Contrast

The nomogram for predicting 3- and 5-year PFS was established by integrating selected one clinical, three CT radiomics, and one PET radiomics features (Fig. 5A and Figure S3A). As seen in Fig. 5B-D and Figure S3B-D, the calibration curves of the nomograms for 3- and 5-year PFS showed good agreement between predicted and actual PFS probabilities across all 3 cohorts.

**Fig. 6:**
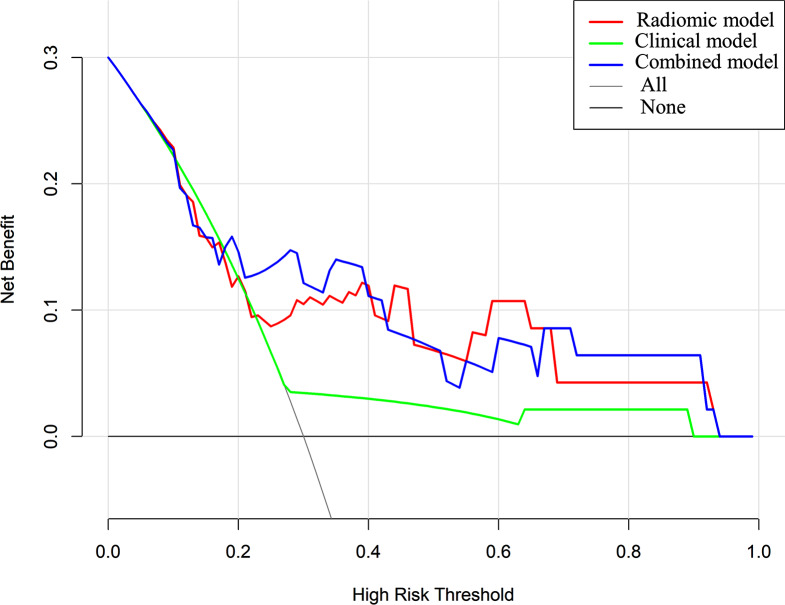
The decision curve analysis (DCA) of prediction models predicting 3-year PFS in training cohort. The X-axis represented the threshold probability that was where the expected benefit of treatment was equal to the expected benefit of avoiding treatment. The Y-axis represented the net benefit. The gray line represents the assumption that all LACC patients gained substantial benefit after CCRT. The horizontal black line represents the assumption that no LACC patients gained substantial benefit after CCRT

DCA was performed to determine the clinical utility of the nomogram (Fig. 6 and Figure S4). The findings also showed that the combined model outperformed the others in terms of accuracy and efficacy.

## Discussion

In this study, we successfully developed a combined risk stratification model that incorporated clinical, PET radiomic, and CT radiomic features for individual prediction of 3- and 5-year PFS probability in patients with LACC who received definitive CCRT. The findings were further validated in an external validation cohort, and an easy-to-use nomogram was developed to aid clinical decision-making.

Several studies have demonstrated that combining radiomics and clinical features may enhance predictive performance for prognosis and therapeutic effects in cervical cancer [23, 24]; these findings are supported by the results of our study. A previous study using radiomics found older age, as defined by an age of 55 years or more, to be an adverse prognostic factor [25]. Our data demonstrated no significant correlation with age; this is in agreement with the findings from another study [26]. Clinical studies have found the pre-treatment neutrophil-to-lymphocyte, platelet-to-lymphocyte, and lymphocyte-to-monocyte ratios to be prognostic indicators [27–29]. However, these studies did not demonstrate any statistically significant association between these ratios and PFS on univariate Cox regression. It is therefore necessary to define more optimal thresholds using an appreciable sample size. Clinical prognosis is most commonly predicted using FIGO staging. In this context, Wei et al. [30] used the FIGO stage and radiomic features to assess survival in patients with LACC. Mu et al. [31] and Jiang et al. [32] incorporated pelvic lymph nodes metastasis status and other clinical factors into radiomics models to improve the predictive value. However, we did not include two recognized prognostic factors (namely, FIGO stage and pelvic lymph nodes metastasis) for model construction, as these variables did not demonstrate significant correlation with PFS on multivariate Cox regression; this could be attributed to the relatively small number of patients. In the present study, pathological type was the only clinically significant feature predictive of PFS. Addition of this feature to the radiomics model enhanced predictive power; this is consistent with findings from previous research [33].

Notably, ^18^F-FDG PET metabolic parameters including SUV_max_, SUV_mean_, MTV, and TLG have unclear prognostic value in cervical cancer. In this context, the pre-therapeutic SUV_max_ of the primary tumor has been reported to correlate significantly with OS and event-free survival in patients with LACC who receive CCRT; it may therefore serve as a key prognostic predictor [34]. In their study, Calles-Sastre et al. [35] found the pre-treatment TLG and MTV to be independent prognostic factors for OS and recurrence-free survival in patients with advanced cervical cancer patients who underwent definitive CCRT; these variables were better than the widely-used parameter, SUV_max_. The performance of the radiomics model in predicting PFS could be improved by incorporating MTV or TLG, as selected using univariate and multivariate Cox analysis [36, 37]. However, none of the parameters, including SUV_max_, SUV_mean_, MTV, and TLG have been used for clinical decision-making in primary cervical tumors [38]. Although univariate analysis indicated TLG and MTV to have predictive value for PFS in this study, this was not found on multivariate analysis. In addition, neither the SUV_max_ nor SUV_mean_ showed prognostic value; this may be attributed to central necrosis of tumor tissue and interference by the inflammatory response. Our results were similar to those observed by Chen et al. [33], in that the ^18^F-FDG PET metabolic parameters did not demonstrate independent prognostic value.

In a study where PET/CT-based radiomics was used to construct prediction models, 5 CT radiomic, 1 PET radiomic, and 6 clinical features were filtered. The radiomics model achieved better predictive performance in the training and internal validation datasets than in the clinical model [38]; this is in agreement with our results. We successfully extracted 4 radiomic features to predict PFS; these included 3 features derived from CT and 1 feature extracted from PET. The number of selected radiomic features derived from PET images was less than that obtained from CT images. This may indicate that CT images contain more prognostic information than PET images, or that PET radiomic features are more likely to be affected by scanning protocols and reconstruction parameters than CT radiomic features [39]. Notably, the maximum 2D diameter (row), as a shape feature, was included in the CT-derived features; this feature characterized the longest distance between tumor surface mesh vertices in the sagittal plane. Nevertheless, the tumor diameter showed no statistically significant association on univariate Cox regression in our study. This shows that radiomics is more precise than manual characterization. In addition, the 4 extracted radiomics features included one wavelet, one logarithm, and two original features. The wavelet and logarithm features could reflect tumor spatial heterogeneity in multiple scales; this suggests that more prognostic information may be mined via filter transformation of the original images. This further reflects the advantages of using radiomics; it offers valuable mined high-dimensional data that are difficult to sense manually. The gray-level non-uniformity (GLNU) feature represents the variability of gray-level intensity values in the image; higher values indicate greater heterogeneity in intensity values. This is in agreement with the findings from a previous study that suggested GLNU to be a poor prognostic marker for cervical cancer [40]. In our previous study using CT radiomics, we extracted maximum 2D diameter (row) and gray level size zone matrix derived from GLNU values to predict PFS in patients with esophageal squamous cell carcinoma [41]. In their study, Lucia et al. [42] found GLNU derived from gray-level run length matrix to be the only PET feature predictive of disease-free survival. The studies differed in that the GLNU was derived from gray-level run length matrix in their study, whereas we derived it from gray level size zone matrix. The radiomic features or models selected and developed in other studies were not fully transposable to our study cohort. Among the 4 features, 3 were textural; this may have helped to exhibit intratumoral heterogeneity and provided additional independent prognostic information [43].

Our models were evaluated in an independent dataset, in which the ROC curve displayed good performance, calibration curves showed good agreement, and DCA confirmed clinical utility; this is one of the strengths of our study. Our study is of clinical value, as is provides a visually quantitative nomogram to aid clinicians in their routine practice. The nomogram combined PET radiomic, CT radiomic, and pathological features, and achieved higher AUC values and better calibration than the radiomics or clinical models alone. In addition, the present study followed the TRIPOD guidelines [44]; this has been outlined in the Table S3.

Despite the favorable results observed using PET/CT-based radiomics, this study has certain limitations. First, it had a retrospective design and offers preliminary findings; in addition, some patients were followed-up for a relatively short duration. Longer follow-up is needed to further evaluate the long-term prognostic value of the established model and nomogram. Second, correlation between human papilloma virus infection and PFS could not be evaluated owing to the absence of complete records pertaining to HPV status in some patients. In addition, some patients lacked data pertaining to the degree of cellular differentiation and expression of serum tumor markers.

In this study, the predictive performance of the clinical model in the external validation cohort exceeded that in the training cohort, which affected the performance of the combined model across different datasets. Our study was conducted at two centers, located in Shandong Province and the Xinjiang Uygur Autonomous Region of China. Shandong is situated along the eastern coastal region, while Xinjiang is located on the northwestern border, close to West Asia. The latest data on cervical cancer in China shows that the incidence rate and mortality rate in the eastern region are higher than those in the western region [45]. Furthermore, the positive HPV infection rates differed between the Uygur and Han in Xinjiang, China, and the genotype distribution of infection was different [46]. The aforementioned limitations may be the key factor in solving this result. Future prospective studies including more clinical parameters and several clinical endpoints need to be performed to further validate and enhance the predictive performance.

## Conclusion

In conclusion, in this multicenter study, we developed and independently validated an effective combined model based on pretreatment PET/CT radiomics and clinical features. The noninvasive nomogram based on the results of the combined model can individually predict PFS in patients with LACC who receive CCRT, and may further provide clinicians with a reference for decision-making.

### Electronic supplementary material

Below is the link to the electronic supplementary material.

Supplementary Material 1: Supplementary figures and tables

## Data Availability

The data are available from the corresponding author on reasonable request.

## Abbreviations

^18^F-FDG: ^18^Fluorine-fluorodeoxyglucose
PET: positron emission tomography
CT: computed tomography
LACC: locally advanced cervical cancer
CCRT: concurrent radiochemotherapy
OS: overall survival
FIGO: the International Federation of Obstetrics and Gynecology
PFS: progression-free survival
MTD: maximum tumor diameter
LNM: lymph node metastasis
ROI: region of interest
SUVmax: maximum standardized uptake value
SUVmean: mean standardized uptake value
SUVmin: minimum standardized uptake value
MTV: metabolic active tumor volume
TLG: total lesion glycolysis
ROC: the receiver operating characteristics
DCA: decision curve analysis
LASSO: the least absolute shrinkage and selection operator
GLNU: gray-level non-uniformity
EBRT: external beam radiotherapy
LMR: lymphocyte to monocyte ratio
PLR: platelet to lymphocyte ratio
NLR: neutrophil to lymphocyte ratio
